# Small round blue cell tumours: diagnostic and prognostic usefulness of the expression of B7-H3 surface molecule

**DOI:** 10.1111/j.1365-2559.2008.03070.x

**Published:** 2008-07

**Authors:** A Gregorio, M V Corrias, R Castriconi, A Dondero, M Mosconi, C Gambini, A Moretta, L Moretta, C Bottino

**Affiliations:** 1 Department of Pathology, University of GenoaGenoa, Italy; 2 Laboratory of Oncology, Gaslini Institute, University of GenoaGenoa, Italy; 3 Department of Experimental Medicine, University of GenoaGenoa, Italy; 4 Centro di Eccellenza per le Ricerche Biomediche, University of GenoaGenoa, Italy; 5 Laboratory of Experimental and Clinical Immunology, Gaslini InstituteGenoa, Italy

**Keywords:** B7-H3, diagnosis, neuroblastoma, small round cell tumours

## Abstract

**Aims:**

To assess whether the expression of B7-H3 surface molecule could improve differential diagnosis of small cell round tumours.

**Methods and results:**

One hundred and one well-characterized paraffin-embedded small round cell tumours, stored in the pathology archive of the Gaslini Institute, were immunohistochemically analysed with the 5B14 monoclonal antibody, which recognizes the surface molecule B7-H3. All lymphoblastic lymphomas and the blastematous component of Wilms’ tumours were completely negative and a few Ewing's sarcoma and Burkitt's lymphoma specimens showed focal positivity, whereas 74% of neuroblastomas, 67% of rhabdomyosarcomas and 100% of medulloblastomas were positive. The pattern of immunoreactivity of 5B14 mAb observed in rhabdomyosarcoma, neuroblastoma and medulloblastoma specimens was limited to the cytoplasmic membrane, and in neuroblastomas areas of rosette formation or of ganglion differentiation were preferentially stained. Interestingly, in neuroblastoma patients high expression of the antigen recognized by the 5B14 mAb was associated with a worse event-free survival.

**Conclusions:**

The 5B14 mAb represents an additional tool for the differential diagnosis of small round cell tumours and might be useful in identifying neuroblastoma patients at risk of relapse who may take advantage of more careful follow-up.

## Introduction

Small round blue cell tumours of childhood include neuroblastoma (NB), rhabdomyosarcoma, non-Hodgkin's lymphoma, Ewing's sarcoma and the closely related primitive neuroectodermal tumour (PNET) and the blastemic component of Wilms’ tumour.^1^ The tumours have similar appearance by light microscopy and are often indistinguishable by common immunocytochemical markers. Moreover, diagnosis, which is often based on the clinical features of the tumours,^2^ may be difficult for those presenting in an unusual clinical context.

For differential diagnosis a panel of antibodies that recognize various tumour-associated markers is currently used. These include neuron-specific enolase (NSE) and GD2, which are present in NB and sometimes in osteosarcomas and rhabdomyosarcomas;^2^,^3^ CD99 expressed by Ewing's sarcoma and by some PNETs;^4^,^5^ NB84 expressed by NB cells and sporadically by Ewing's and PNETs;^6^ and desmin and cytokeratin present in desmoplastic tumours.^7^ However, none of these markers has, by itself, clinical utility in unambiguously differentiating small round blue cell tumours.^8^

We recently isolated a novel monoclonal antibody termed 5B14,^9^ which specifically recognizes a surface glycoprotein termed B7-H3. This molecule is an additional member of the B7 family, which also includes B7-1 (CD80) and B7-2 (CD86).^10^ Unlike most B7 members, whose receptors have been identified, B7-H3 represents an orphan ligand. On the other hand, functional data suggest that T and natural killer (NK) lymphocytes express specific receptor(s) displaying either an inhibitory or co-stimulatory function.^11^,^12^ In particular, B7-H3 expressed at the tumour cell surface exerts a protective role in NK-mediated lysis.^9^ Notably, the mAb selectively stains NB cells infiltrating the bone marrow of stage 4 patients.^13^

Differentiating a blue cell tumour from others is a diagnostic challenge when considering that both treatment and prognosis vary greatly among these tumours. In this study we evaluated the potential use of 5B14 mAb for diagnostic and/or prognostic purposes. We analysed the expression of B7-H3 in a large number of paraffin-embedded small round blue cell tumours. Tumour specimens included lymphoblastic and Burkitt's lymphomas, blastemic component of Wilms’ tumour, primary NB, rhabdomyosarcomas and medulloblastomas.

## Material and methods

One hundred and one previously diagnosed small round blue cell paraffin-embedded tumours, stored in the Department of Pathology of the Gaslini Institute, were analysed. These included seven lymphoblastic lymphomas, 11 Burkitt's lymphomas, eight Wilms’ tumours, six PNET/Ewing's sarcomas, nine rhabdomyosarcomas, seven medulloblastomas and 53 NB. All NB tumours were schwannian stroma poor with different differentiation grades and different mitotic karryoretic indices (MKI); 15 were stage 1, 11 stage 2, one stage 3, 20 stage 4 and six stage 4s, as assessed according to International Neuroblastoma Staging System criteria.^14^ Only four of the 53 NB specimens were *Myc-N* amplified.

### Immunohistochemistry

5B14 mAb (anti-B7H3, IgM) was obtained by immunizing a 5-week-old BALB/c mouse with the ACN human neuroblastoma cell line, as previously described.^9^

Formalin-fixed paraffin-embedded tissue sections were de-paraffinized, rehydrated through graded ethanol solutions and treated in 3% H_2_O_2_ to block endogenous peroxidase. Immunohistochemical labelling was performed by a three-step indirect immunoperoxidase technique. Once hydrated, sections were heated for 30 min at 99°C in citrate buffer solution, pH 6.0 (Dako, Glostrup, Denmark) and incubated overnight at 4°C with a 1:2000 dilution of purified 5B14 mAb (0.5 mg/ml). After washing, sections were incubated for 30 min at room temperature with anti-mouse antibody conjugated to peroxidase-labelled dextran polymer (Dako). After washing, the slides were incubated with the diaminobenzidine substrate at room temperature. Slides were counterstained with Mayer's haematoxylin. Negative controls, consisting of slides incubated with mouse normal serum (X0910; Dako), were always run simultaneously.

### Grading analysis

The immunohistochemical results were classified using two different systems. With one system reactivity was qualitatively scored as 0 in the absence of reactivity; one in the presence of weak and partial membranous reactivity in >10% of cells; two when moderate membranous reactivity was detected in >10% of cells; and three when intense membranous reactivity occurred in >10% of cells. With the second system, reactivity was graded semiquantitatively as: ± with 10–25% positive tumour cells; + with 25–50% positive tumour cells; ++ with 50–75% positive tumour cells; and +++ with 75–100% positive tumour cells.

### Survival and statistical analysis

Clinical data of NB patients were retrieved from the Italian neuroblastoma registry, which collects information on clinical and biological characteristics of patients at diagnosis and during their front-line treatment.^15^ Survival curves were constructed by using the Kaplan–Meier method, and the generalized Wilcoxon log-rank test (Peto) was used to compare the curves. A *P*-value <0.05 was considered to be statistically significant. Statistical analyses were performed using the Statsdirect software (Statsdirects Ltd, Sale, UK).

## Results

One hundred and one small round cell tumours were analysed by immunocytochemistry with 5B14 mAb (Table 1). All the lymphoblastic lymphomas (seven cases) and blastemic components of Wilms’ tumours (eight cases) were negative. Two out of six Ewing's sarcoma (33%) and three out of 11 Burkitt's lymphoma (27%) specimens were slightly immunoreactive (Table 1). Conversely, 6/9 rhabdomyosarcoma (67%), 39/53 NB (74%) and 7/7 medulloblastoma (100%) specimens were positive. By qualitative analysis, five medulloblastomas and 13 NB scored 3; two medulloblastomas, nine NB and four rhabdomyosarcomas scored 2; 17 NB and two rhabdomyosarcomas scored 1 (Table 1).

**Table 1:** Immunocytochemical analysis of 101 small blue cell tumours with 5B14 mAb

Tumours	*n*	Neg.	(%)	Pos.	*n*	Score*	*n*	Grade†
Lymphoblastic lymphoma	7	7	100	0		–		
Wilms’ tumours	8	8	100	0		–		
PNET/Ewing’s	6	4	66	2				
					0	3	0	+++
					0	2	1	++
					2	1	1	+
Burkitt’s lymphoma	11	8	73	3‡				
					0	3	0	+++
					0	2	0	++
					3	1	1	+
							2	±
Rhabdomyosarcoma	9	3	33	6				
					0	3	0	+++
					4	2	0	++
					2	1	6	+
Neuroblastoma	53	14	26	39				
					13	3	14	+++
					9	2	7	++
					17	1	6	+
							12	±
Medulloblastoma	7	0		7				
					5	3	5	+++
					2	2	1	++
					0	1	1	±

* 0, no staining;

1, weak and partial membranous immunoreactivity in >10% of cells;

2, moderate membranous reactivity in >10% of cells;

3, intense membranous reactivity in >10% of cells.

† 10–25% positive tumour cells;

±, 10–25% positive tumour cells;

+, 25–50% positive tumour cells; ++, 50–75% positive tumour cells; +++, 75–100% positive tumour cells.

‡ Focal positivity.

When semiquantitative analysis was performed, six positive rhabdomyosarcoma specimens, three positive Burkitt's lymphomas, one positive Ewing's sarcoma and one positive medulloblastoma were graded + or ± (Table 1). Conversely, 6/7 medulloblastomas (86%), 21/39 NB (54%) and one Ewing’s tumour were graded +++ or ++. One medulloblastoma and the remaining 18 NB tumours had a lower grading (six samples +, and 12 samples ±) (Table 1).

**Figure 1:**
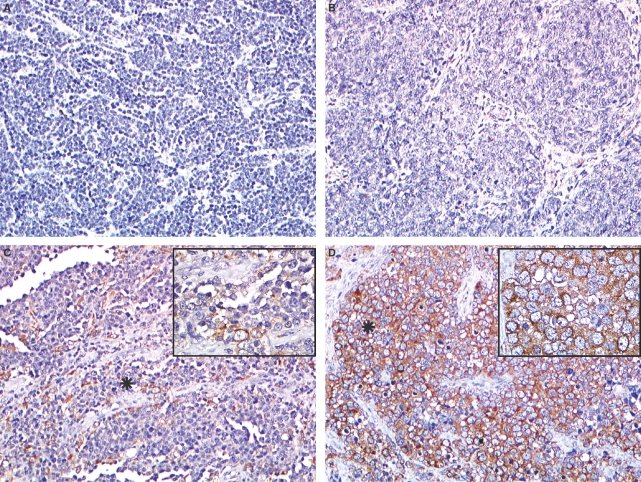
Reactivity of 5B14 mAb in small round blue cell tumours. Lymphoblastic lymphomas (A) and blastematous components of Wilms’ tumours (B) were always negative. Three out of 11 Burkitt's lymphoma specimens showed focal positivity (C) Several rhabdomyosarcomas (D), as well as medulloblastomas (not shown) and neuroblastomas (NB) (see Figure 2), were positive. *Area corresponding to the inset. Original magnification ×100, insets ×400.

**Figure 2:**
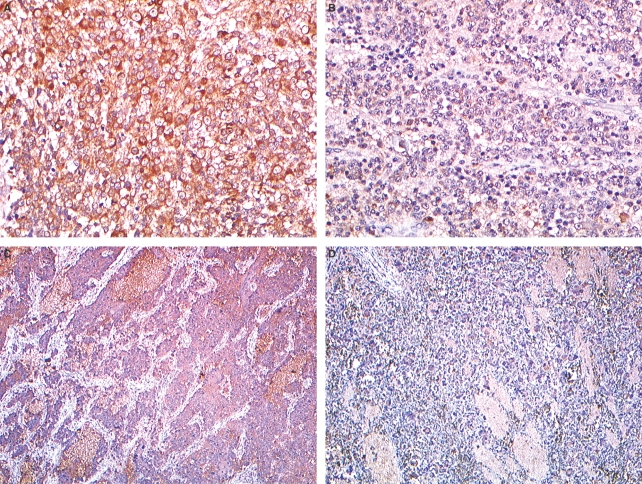
Different score and grade of 5B14 staining in neuroblastoma (NB). Representative NB with score 3 (A), score 1 (B), grade +++ (C) and grade + (D). Original magnification ×100 (A–B), ×40 (C–D).

**Figure 3:**
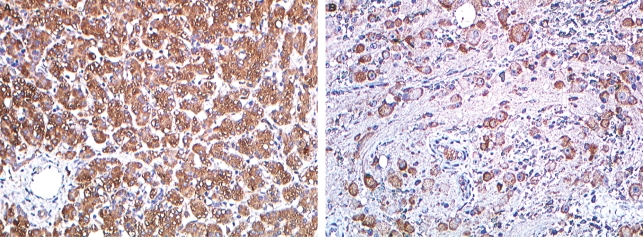
Rosettes of neuroblastoma (NB) cells (A) and differentiating NB cells (B) stained with 5B14 antibody. Original magnification ×100.

As shown in Figure 1, where two representative negative specimens of lymphoblastic lymphoma and Wilms’ tumour are included (panels A and B, respectively), the membranous pattern of immunoreactivity of 5B14 antibody was focal in Burkitt's lymphomas (Figure 1C) and Ewing's sarcomas (not shown). Conversely, intense reactivity was observed in rhabdomyosarcomas (Figure 1D) and medulloblastomas (not shown). In NB specimens, 5B14 immunoreactivity was highly heterogeneous. In addition to 26% of negative samples (Table 1), both the intensity (score) and the percentages (grade) of positive cells varied greatly among the different samples (Figure 2A,B, score 3 and score 1, respectively; Figure 2C,D, grade +++ and grade +, respectively). In NB specimens, however, the 5B14 antibody preferentially stained areas of rosette formation or of ganglion differentiation (Figure 3A, B, respectively).

**Figure 4:**
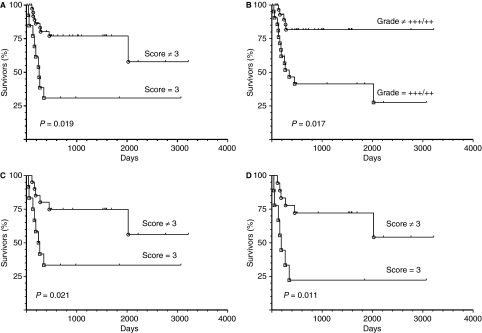
Kaplan–Meyer plots of event-free survival of neuroblastoma (NB) patients stratified according to absence/presence in their tumours of score 3 positivity (all patients, A); grade +++/++ (all patients, B); score 3 positivity (stage 4 patients excluded, C); score 3 positivity (only stage 1, 2 and 3 patients, D).

B7-H3, the antigen recognized by the 5B14 mAb, has been shown to downregulate NK- and/or T-mediated antitumour activity by interacting with a putative inhibitory receptor.^9^ Based on the great heterogeneity of 5B14 reactivity in NB tumours, we sought to perform NB patients’ survival analysis by stratifying them according to the 5B14 positivity in their tumours. Initially, patients were stratified based on the presence or absence of reactivity, but no difference in survival was observed (not shown). However, when patients were stratified according to the presence or absence of high expression of B7-H3 (score 3 or grade ++/+++) in their tumours, a highly significant difference was observed. As shown in Figure 4, patients who scored 3 (Figure 4A) or +++/++ (Figure 4B) had a worse event-free survival than patients that scored <3 or were graded less than ++ (*P* = 0.019 and *P* = 0.0017, respectively). Interestingly, the difference in event-free survival was observed also when high-risk patients (stage 4) were excluded from the analysis (Figure 4C, *P* = 0.021) and when only patients with localized disease (stage 1–3) were considered for analysis (Figure 4D, *P* = 0.011).

## Discussion

We have shown that 74% of NB, 67% of rhabdomyosarcomas and 100% of medulloblastomas were stained by the 5B14 mAb, which recognizes the B7-H3 molecule.^9^ Conversely, 100% of lymphoblastic lymphomas and the blastemic component of Wilms’ tumours were completely negative. Thus, this mAb has a clinical utility in the differential diagnosis of small round blue cell tumours. In particular, it may be a useful tool for tumours presenting in an unusual clinical context, when undifferentiated small blue cells without neural, rhabdoid or epithelial differentiation are observed in light microscopy.

Whereas 100% of medulloblastomas were positive, 5B14 showed a variable and limited specificity and sensitivity for NB, rhabdomyosarcoma and Ewing's tumour, similarly to other commercial mAbs.^6^ For example, the identification of NB cells usually relies on the combination of CD99^4^,^5^ or desmin and cytokeratin negativity^7^ with NB84 positivity,^6^ although skeletal and extraskeletal Ewing's tumours and PNETs may also be NB84+.^6^ Indeed, the anti-GD2 mAbs (GD2 is a NB-associated marker) cannot be used on paraffin-embedded tissue and may be positive in osteosarcoma and rhabdomyosarcoma.^3^ Finally, NSE,^2^,^16^–^18^ synaptophysin^19^,^20^ and neurofilament^21^are also not NB-specific.

Thus, differential diagnosis of NB tumours may take advantage of the combined use of NB84 and 5B14 mAbs. In this context, it is of note that the expression of the (still unknown) NB84-reactive molecule^2^ is limited to the cytoplasm, whereas B7-H3, the antigen recognized by 5B14 mAb, is a transmembrane glycoprotein expressed on the cell surface. In addition, unlike the antigen recognized by the NB84 mAb,^6^ the B7-H3 molecule seems to be expressed by both neural crest- and tube-derived precursors, as demonstrated by the absolute 5B14 positivity observed in medulloblastomas.

The fact that some NB tumours are 5B14− or faintly 5B14+ confirms the great heterogeneity of this malignancy^22^ and suggests that in some NB neuronal markers could be down-regulated. The finding that high expression of B7-H3 in NB is associated with a worse event-free survival merits further investigation. This correlation, in fact, was also observed in patients with localized disease (stage 1–3) and in those with metastatic disease <1 year old (stage 4s). It is of note that these patients generally have a good survival,^23^ and few of them relapse and eventually die of the disease. For these patients, *Myc-N* amplification is the only independent factor of bad prognosis.^24^,^25^ In our study, only four out of 53 NB tumours were *Myc-N* amplified. Thus, the fact that high reactivity of the 5B14 mAb was associated with a worse prognosis supports the tenet that high expression of B7-H3 surface molecule, together with other factors such as age and MKI, may help to identify patients at risk of relapse who may take advantage of more careful follow-up.

## Abbreviations:

MKI: mitotic karryhoretic index
NB: neuroblastoma
NK: natural killer
NSE: neuron-specific enolase
PNET: primitive neuroectodermal tumour
